# Association between Dysbiosis in the Gut Microbiota of Primary Osteoporosis Patients and Bone Loss

**DOI:** 10.14336/AD.2023.0425

**Published:** 2023-12-01

**Authors:** Julien D.H. Waldbaum, Jessica Xhumari, Oluwamayowa S. Akinsuyi, Bahram Arjmandi, Stephen Anton, Luiz Fernando Wurdig Roesch

**Affiliations:** ^1^Department of Microbiology and Cell Science, College of Agriculture and Life Sciences, University of Florida, Florida, USA.; ^2^Department of Nutrition and Integrative Physiology, College of Health and Human Sciences, Florida State University, Florida, USA.; ^3^Department of Physiology and Aging, College of Public Health and Health Professions, College of Medicine, University of Florida, Florida, USA.

**Keywords:** Next-Generation-sequencing, microbiome, 16SrRNA, dysbiosis, aging, osteoporosis

## Abstract

In recent decades, gut microbiome research has experienced significant growth, driven by technological advances that enable quantifying bacterial taxa with greater precision. Age, diet, and living environment have emerged as three key factors influencing gut microbes. Dysbiosis, resulting from alterations in these factors, may lead to changes in bacterial metabolites that regulate pro- and anti-inflammatory processes and consequently impact bone health. Restoration of a healthy microbiome signature could mitigate inflammation and potentially reduce bone loss associated with osteoporosis or experienced by astronauts during spaceflight. However, current research is hindered by contradictory findings, insufficient sample sizes, and inconsistency in experimental conditions and controls. Despite progress in sequencing technology, defining a healthy gut microbiome across global populations remains elusive. Challenges persist in identifying accurate gut bacterial metabolics, specific taxa, and their effects on host physiology. We suggest greater attention be directed towards this issue in Western countries as the cost of treating osteoporosis in the United States reaches billions of dollars annually, with expenses projected to continue rising.

## Background

The human microbiome is a system of bacteria, archaea, eukaryotes, and viruses found in and on the body that works in symbiosis with the host [1]. These microbes can impact the body’s physiology, promoting health or disease based on their richness, diversity, and interactions. In this literature review, we are interested in how contributions such as microbes educating the immune system and regulating inflammatory metabolisms can provide insight into bone loss. The gut microbiome, which consists of about 1200 species of bacteria [2], represents the most diverse microbial community on the human body. Compiled data from the MetaHit (www.gutmicrobiotaforhealth.com/metahit/) and the Human Microbiome Project (https://hmpdacc.org/) have identified that 93.5% of the human gut microbiome consists of *Bacteroidetes*, *Firmicutes*, and *Actinobacteria* [3]. Variations in gut microbes among healthy individuals from different backgrounds constitute a challenge to creating a baseline for what is considered healthy. Our ability to simply survey the gut microbiome has also been difficult. A decade ago, knowledge about the gut microbiome emerged from labor-intensive culture-based methods. It is only recently that culture-independent approaches such as high-throughput and low-cost sequencing methods which focus on 16S rRNA gene sequencing of nine highly variable regions (V1-V9) of the 16S rRNA gene present in all bacteria [3] have allowed researchers to obtain patterns of microbial populations in the body. Using these techniques, we can sample fecal bacteria from different communities [1] and analyze shorter subregions of the 16S rRNA gene in greater depth, unlocking novel and uncharacterized microbes [3]. The gut microbiome bacteria participate in the human body's physiological functions, such as nutrient production and absorption, metabolic balance, immune function, brain behavioral function, and inflammatory response [4]. This microbiome is also associated with human diseases such as type 1 and type 2 diabetes, Parkinson’s disease, and rheumatoid arthritis, among others [5].

Within this work, we attempted to better understand gut dysbiosis and its association with primary osteoporosis, which consists primarily of bone loss and inflammation in the body [6]. Healthy bone density is maintained by regulated metabolisms of bone formation mediated by osteoblasts and bone resorption mediated by osteoclasts. These processes are repeated, coupled, and maintained [7]. The factors that regulate this coupled reaction have been suggested to be directly induced by cell-cell contact and cytokines [7]. Therefore, any destabilization of the cytokine regulation could be crucial in causing bone defects, and this change is perhaps accentuated by gut microbiome dysbiosis. Osteoporosis is the most common bone disease [8]. The resulting bone fragility and microstructural damage can lead to osteoporotic fractures, which may occur during daily activities. These fractures are a leading cause of disability and death among elderly individuals with the condition [8], as well as institutionalization and economic costs [9]. In the United States, the cost of osteoporosis amounted to $57 billion in 2018, which is expected to nearly double by 2040 [9]. Thus, exploring additional potential causes of osteoporosis, a non-reversible and incurable disease, is an engrossing area of research.

## Factors that alter the gut microbiota

### Aging

Modern improvements in our capacity to precisely identify the gut microbiome, although not yet perfectioned, have led to several studies proposing that the gut microbiome changes over time as people age [10]. Without other factors, fecal microbiota scarcely changes during adult life [10]. However, there is a rapid change in the gut microbiome during early childhood (ages 0-3) and a gradual change in older populations (65+ years) [11]. Despite recent progress in understanding the gut microbiome composition, our comprehension of when age-associated changes happen and what host physiology they reorganize remains unfamiliar [11]. Nevertheless, increasing number of studies from Eastern countries are emerging on the topic, further emphasizing the significance of this research area in Western contexts. By understanding how the gut microbiome changes with age, we can potentially determine its contribution to geriatric diseases such as osteoporosis.

In a study of 371 human subjects ranging from 0-104 years of age, using a UniFrac principal coordinate analysis of 5,952 operational taxonomic units classified into 187 bacterial genera, Odamaki et al. [12], determined the extent of similarity between microbial communities. Age explained the variations in data, and no gender differences were observed. Similarly, Biagi et al. [13] conducted a characterization of fecal samples from centenarians, elderly, and young adults performed using HITChip Hybridization. After performing a redundancy analysis (RDA) of the gut bacteria in all three groups, the researchers repeated the experiment, excluding males. The restriction did not change the overall shape of the RDA plot, suggesting that gender differences did not influence the effect of aging on the gut microbiota composition [13].

According to Odamaki et al. [12], *Actinobacteria*, which are suspected to modulate gut permeability, the immune system, and gut-brain axis [14]; decreased substantially after 2-4 years of age and continued to decrease with increasing age. *Firmicutes* such as *Faecalibacterium prausnitzii*, which produce butyrate to keep the colon healthy and extract energy from food [15], was the most abundant phylum after 2-4 years of age and less predominant before 4 years of age. Relative abundance of *Bacteroidetes*, which are major players in sustaining the microbial food web of the gut [16], was stable throughout the samples until subjects reached 70 years of age when an increase was reported [12]. High levels of *Bacteroidetes* have been associated with reduced digestive capacity and constipation, indicating that a healthy gut is sensitive to the quantity of each bacterium even if they are qualified as beneficial to the host. Furthermore, by clustering the observed genera into co-abundance gene (CAG) groups, compelling differences in microbiota between the subject groups of 0-104 years of age were presented. The study showed that there was an age-related reduction of the genus *Bifidobacterium*, which is a bacterium that down-regulates pro-inflammatory responses in the gut [12]. This finding could explain the contribution of gut microbiome dysbiosis and inflammatory responses to diseases in people of advanced age. A growing body of literature on mutualism among species has shown that there could be an issue when multiple genetically different organisms interact with the host as they cannot grow well under the same environmental conditions [17]. Such was the case with *Bifidobacterium* populations reported to be stimulated efficiently with a decrease in *Enterobacteriaceae*. *Enterobacteriaceae* increased with age in the study, creating a greater environmental challenge for the weakened intestinal barrier, increasing the inflammatory response [12]. Therefore, positive and negative relationships between gut bacteria can be established.

According to Biagi et al. [13], a shift in the dominant species within several bacterial groups was observed, as well as an increase in facultative anaerobes, with no difference in the abundance of anaerobic bacteria. After analysis, the gut microbiota of the first cluster of subjects containing 67% of centenarians had a higher amount of *Proteobacteria* and *Bacilli* than two other clusters of subjects with similar compositions of young, elderly, and centenarian patients. These 2 mixed clusters showed a lower abundance of *Bacteroidetes* than those with centenarians [13]. The researchers proposed that the decrease in *Bacteroidetes* in the gut microbiota is not age-related, which contradicts the study by Odamaki et al. [12]. The differences between the *Firmicutes/ Bacteroidetes* ratio were not significant when comparing individual clusters of young, elderly, and centenarian subjects [13]. This study delivers more insight into gradual changes in gut microbiota in older populations. In fact, hierarchical clustering of genus-like taxa in a heat map of HITChip profiles of the subjects showed that centenarians formed a separate group while young and elderly subjects overlapped. After Simpson indices were obtained using the Simpson reciprocal index of diversity, Biagi et al. [13], found that the microbiota of centenarians was much less diverse than that of elderly and young adults. In contrast, Simpson indices of elderly and young adults were similar. Centenarians have a unique microbial composition, and perhaps it is what enables them to live longer and get the right amount of nutrients from a functional GI tract. This differential microbiota could also limit inflammation compared to the elderly population.

A study by Saraswati and Sitaraman [18], states that the most noticeable change in the gut microbiome between a young and elderly person is the relative abundance of *Bacteroides* and *Firmicutes*. The paper suggests that *Bacteroides* decrease with age and higher levels of *Firmicutes* are found in young people. Therefore, this study proposes that *Bacteroides* decrease with age, an opposite result to the study from Odamaki et al. [12], which found that *Bacteroides* levels are stable and increase once a patient reaches old age (70 years +). The results from Saraswati and Sitaraman [18] are plausible based on the function of *Bacteroides* and *Firmicutes* in the body described earlier. It is possible that with age, a lower number of *Bacteroides* and a reduced number of *Firmicutes* could weaken digestive capacity and induce constipation, which are usual challenges that older people must face. However, there is also a possibility that an over-abundant number of *Bacteroidetes* in the gut have the same harmful effect, reinforcing the idea that particular (but not singular) gut bacteria diversity is required to stay healthy.

Here we showed that there are 3 published studies in a span of 10 years presenting 3 different results on gut bacteria changing with age. Such an outcome could indicate that the area of research is not yet fully understood and disparate results are coming from different experiments. Obtaining reliable results on the gut microbiome could provide considerable progress in anti-inflammatory treatments and gut health. There must be more concentrated efforts in studying the gut microbiome.

### Diet

Since people have differing diets, they can harbor different gut microbiomes. Two distinctly diversified gut microbiomes can be considered healthy and beneficial to the host. This information makes setting a baseline difficult when establishing healthy gut bacteria quantity and diversity. Thus, researchers can use an animal model system, where host genetics and diet can be highly controlled. Although findings on animals cannot always translate to humans and confirm a clinical outcome, they can provide insight into microbiome evaluation. In 2011, a study involving human subjects showed that altered nutrient load induced rapid changes in the bacterial composition of the human gut [19]. The stool energy loss determined that an increase in *Firmicutes* and a corresponding decrease in *Bacteroidetes* was associated with an increase in harvested energy by at least 150 kcal. Recent studies demonstrated that the relationship between gut bacteria and obesity in humans is more complex than the ratio of bacteria. The interplay of these bacteria should be studied in connection with all gut bacteria that thrive in a similar environment. Nonetheless, the study suggested that epithelial MyD88 and RegIIIγ were key host components regulating the intestinal gut microbiota [20]. The association between the immune system and host-microbial spatial interactions is important when analyzing what promotes inflammation due to gut dysbiosis.

It can be challenging to monitor an individual's diet and nutritional status as it requires a comprehensive understanding of their dietary habits over an extended period. Thus, databases from many organizations are searched to obtain a pattern of food consumption. Using 16S rRNA gene sequencing, UniFrac clustering, and CAZyme analysis, a metagenomics study of gut bacteria composition in lean and obese pairs of twins across the USA has associated a decreased diversity of gut bacterial species with obesity [21]. It is believed that low gut microbiome diversity could affect the functioning of the bacterial community and weaken the immune system [22]. For future reference, an enzyme called a CAZyme in the database is specialized in building and breaking down complex carbohydrates and glycoconjugates [23]. The gut is a competitive environment for nutrition, and bacteria use these enzymes to gain a competitive advantage to thrive over other bacteria [24]. Although humans lack the necessary enzymes to fully digest dietary fiber, the indigestible component of plant foods, gut bacteria can metabolize certain dietary fibers and produce energy substrates for the host. This symbiosis involves the host providing the microbially accessible carbohydrates (MACs) while the bacteria digest them and return 10% of the energy to the host [24].

Diet is a major factor influencing the composition of the host microbiome, and it must be considered when sampling gut bacteria [18]. Only considering age as a factor is insufficient. The study by Odamaki et al. [12], had 371 Japanese participants, and the study by Biagi et al. [13] and Saraswati and Sitaraman [18] did not specify the background of their participants. People from different cultural backgrounds have diverging diets. A Western diet could be considered as high-fat and sugar-rich, contributing to a *Bacteroides*-dominant environment [18]. An Oriental diet could be considered a high-fiber diet, contributing to a *Firmicutes*-dominant environment. Therefore, if diets are consistent, features of the gene content of gut communities are shared among family members and transmitted to generations [21]. A demonstration of this claim can be found in a study by Hehemann et al. [25]. Β-porphyranase is a gene to degrade seaweed-associated glycans in marine microbes such as *Zobellia galactanivorans*, a marine *Bacteroidetes* [22]. These microbes are also associated with non-sterile foods consumed by the Japanese population. Since CAZymes are absent in the human genome but present in gut microbes, researchers wondered whether the Japanese population consuming seaweed as an important component of their diet would also have this CAZyme. Then, it was found that the gut bacterium *Bacteroides plebeius* must have obtained β-porphyranase by horizontal gene transfer [25], enabling the host of the bacterium to digest the glycans easily and extract greater energy from the food. It is believed that *B. plebeius* acquired the porphyran utilization locus from an ancestral porphyranolytic bacterium that is related *to Z. galactanivorans*. Interestingly, *Bacteroides plebius* is not prominently represented in the gut microbiomes of North Americans but was present in the gut of the Japanese subjects [22]. Finally, the results of this study are significant because it is believed that changes in diet patterns could lead to switches in the diversity of the gut microbiome, leading to consequences such as a weakened immune response and diminished nutrition gathered by the host. Different bacterial species in the human gut are sensitive to the proportions and diversity of ingested food.

Conducting research on how diet affects the human gut microbiome has the potential to identify the most effective bacteria and establish a baseline of bacterial diversity and volume for a healthy gut. Hence, this research would have significant implications for improving the health and lifestyle of aging individuals suffering from a weakened skeletal structure and inflammatory pain. Instead of relying on over-the-counter anti-inflammatory pills or prescription drugs to treat osteoporosis, individuals may be able to prevent or reduce symptoms by treating their gut microbiota with a specialized diet that meets recommended standards.

### Environment

Living systems are strongly impacted by physical factors such as temperature, pH, and the concentration of compounds in the environment. This is true whether considering the extinction of a species of animals due to environmental factors or considering a physical disturbance in the gut microbiome that could have long-term consequences [26]. From metagenomic studies, it is believed that the environment during early postnatal growth plays a major role in structuring the gut microbiome of a person in their adult life [27]. Similarly, the environment a person lives in is suspected to influence the diversity of the gut microbiome. For example, there has been a lot of research done on how modern urban living affects human health, perhaps interconnected with industrialization and access to modern drugs such as antiacids, laxatives, and anti-inflammatory pills. However, there is still insufficient data to understand the role of the gut microbiome in dealing with factors of urbanization. Nonetheless, an example of this claim may be that the effects of rapid urbanization could likely be reflected in the gut microbiome, and this alteration could lead to inflammation and disease [28]. There is no direct evidence of how urbanization affects the human gut and inflammation. There is a large volume of data that has been compared between healthy individuals in non-Western urban and rural areas. The outcome of this comparison is that populations residing in a non-Western/rural area have a higher bacterial diversity than urban populations in the West or Europe [28]. For instance, fecal samples were taken from children living in a rural African village near Burkina Faso. Their diet resembled that of early agriculture and was high in fiber. When the samples were compared with children living in the city of Florence in Italy, an enrichment of *Bacteroidetes* and a depletion of *Firmicutes* was observed in the African children. These results contrast with the enrichment of *Firmicutes* typically observed in Eastern high-fiber diets. Additionally, there was a unique abundance of bacteria from the genera *Prevotella* and *Xylanibacter*, which contain genes to hydrolyze cellulose and xylan [28]. These bacteria were deficient in all the European children studied [28].

The redundant difficulty of establishing a baseline for a healthy gut is thus further illustrated. There is much opposition and disunity in results concerning the gut microbiome, and unified results are not yet collected. In the study by Zuo et al. [28], the environment and age of the African subjects could have influenced the ratio of *Firmicutes* when compared to the ratio of previously observed Japanese subjects. Thus, the direct role of the environment in this study remains unclear.

On a micro-scale, the gut environment is determined by physical factors, including osmolality, pH, and mucus stiffness. These parameters are highly regulated by our epithelial cells and vary along the length of the GI tract [26]. The environment and physical factors that impact bacteria have been researched, just as the impact of metabolites on a host have been researched. The interdependence between the two may be overlooked, and the likelihood of the importance of physical factors as a driver of community composition is currently being explored [26]. Glucose has biochemical properties for the bacteria in our gut, but it also affects the osmotic pressure of the area [26]. Similarly, short-chain fatty acids (SCFA) are important metabolites for microbe growth, and they also affect gut pH and hydrogen potential [26]. Through fermentation products and excretion of SCFA, bacteria are also able to contribute to their habitat in the gut. As mentioned previously, bacteria are constantly competing for nutrition in the gut, and they require specific environmental conditions to thrive. Specialized niches along the intestine with distinct environmental conditions harbor different bacterial species with precise preferences. By the time stool samples are excreted and collected, the rich history of the microbes’ niche environment in the intestine, as well as the microbes’ impact during the journey through the intestinal tract, is lost. Important aspects of the host’s environmental contributions to the gut microbiome are dismissed from the analysis. Thus, another challenge arises in unifying results and understanding which physical conditions and which bacteria could contribute to inflammation and bone loss in diseases such as osteoporosis.

### Prebiotics

Gut bacteria absorption of nutrients that promote bone formation has been linked to probiotic and prebiotic treatments alongside an increase in magnesium and phosphorus availability which are essential for bone development and mineralization [29]. Prebiotics are a useful product for driving changes in pre-existing gut bacteria. Unlike probiotics, prebiotics do not provide bacterial cultures but instead nutrients that enhance the growth and activity of the existing microbial community. This approach enables researchers to investigate the gut microbiome’s response to increased nutrient ability without introducing new bacterial species and thereby altering the system’s composition. Some of the most beneficial prebiotics for humans are fructo-oligosaccharides and galacto-oligosaccharides. Still, they are found in low quantities in food, and scientists are trying to produce prebiotics on an industrial scale [30]. In a study by Abrams et al. [31], one year supply of an insulin-type fructan prebiotic was administered to adolescents. The effects on calcium absorption were reported at baseline, after eight weeks, and after a year. Calcium absorption, whole-body bone mineral content, and whole-body bone mineral density were significantly greater in the adolescents who received the prebiotic treatment versus the control group.

Similarly, an older study by Ohta et al. [32] already noticed that administering a fructo-oligosaccharide prebiotic in rats increased the absorption of calcium, magnesium, and water in the colon. This was noticed by the linear disappearance of calcium and magnesium during their passage in the colon. Fecal contents were processed, and it was also found that indigestible and fermentable carbohydrates facilitate this absorption by being metabolized into SCFA metabolites by gut bacteria. Although these two older studies do not bring about any specificity to which mechanisms cause this increase in calcium absorption, they demonstrated that healthy and thriving gut bacteria contribute to the absorption of more bone-related nutrients, facilitating prosperous bone mineralization and formation. Additionally, the gut microbiota enhances the metabolism of B group vitamins and K vitamin better when administered prebiotics, and these vitamins are necessary for the process of absorbing calcium [21].

### Probiotics

Probiotics are live microorganisms, commonly referred to as beneficial bacteria, that can be utilized to alleviate gut-related diseases such as osteoporosis when consumed in sufficient quantities [33]. Common bacteria and yeast used as probiotics include *Lactobacillus, Bifidobacterium, Bacillus, Enterococcus*, and *Saccharomyce*s. Probiotics work by restoring the composition and functions of the gut microbiome. They achieve this by directly interacting with the existing microbiota, inhibiting the growth of pathogenic bacteria through the production of antimicrobial agents, and competing for nutrients and binding sites on the intestinal mucosa [34, 35].

Considering the positive impact of probiotics on the gut-bone axis, several authors have proposed probiotics as potential adjuvants to calcium and vitamin D for promoting bone health [36]. Numerous studies have demonstrated that probiotics reduce bone loss by altering the composition of the gut microbiome [37, 38]. A recent study by Zhao et al. [39] revealed that administering *Bifidobacterium *lactis probiotics attenuated bone loss in post-menopausal patients by modulating the gut microbiota, leading to corresponding changes of calcium regulators such as vitamin D and parathyroid hormone in serum [39]. Specifically, the level of vitamin D, a positive regulator of calcium, increased while the parathyroid hormone, a negative regulator of calcium, decreased. Another study conducted by Sign et al. [40], reported that probiotics, encouraged the growth of beneficial bacteria such as *Bifidobacteria *and *Lactobacillus* species while decreasing the abundance of *Clostridia* species [40]. Notably, *Bifidobacteria *and *Lactobacillus* have been reported to positively correlate with bone mineral density (BMD) [41].

Probiotics are also reported to attenuate age-related bone loss by increasing the relative abundance of SCFA producing bacteria in the gut, which was reflected by the significantly increased concentration of SCFA measured in the stool of aged mice that consumed probiotics [42]. The mechanism by which SCFAs reduced bone resorption is closely associated with increased intestinal calcium absorption, osteoblast differentiation, and decreasing osteoclast activity [43, 44]. Similarly, Yuan et al. [45] observed that *Bacteroidetes vulgatus* probiotic treatment restored gut microbial composition and subsequently downregulates the Lipopolysaccharide/TLR-4/p-NF-κB pathway. This led to a reduction of pro-inflammatory cytokines such as TNF-α and Rank, alleviating bone loss in ovariectomized mice model [45]. These findings suggest that modulating the gut microbiome with probiotics has a direct influence on bone health, highlighting the potential of probiotics as a therapeutic strategy for preventing and treating bone loss.

### Association between gut microbiota and bone formation

The human skeleton constantly undergoes degradation and remodeling. Monocyte-myeloid derived osteoclasts remove packets of old bone by resorption, and mesenchymal derived osteoblasts form new bone material. To maintain a healthy skeleton and avoid alteration in bone mass and quality, these mechanisms must be coupled and regulated so that there is no net gain or loss of tissue [46]. Various factors can derail this physiological process, and there could be an association between gut bacteria and bone loss. The gut microbiome, while being essential for immune system maturation and protection in the gut, could also regulate organs through the diffusion of metabolites [47]. For example, Indoleamine-2,3- dioxygenase-1 is a bacterial metabolite that suppresses inflammation through its tryptophan degradation pathway. The first breakdown product, L-kynurenine, activates the aryl hydrocarbon receptor (Ahr) in lymphoid cells and promotes the production and development of regulatory T cells (Tregs) [48]. In turn, these Ahr ligands also promote interleukin-22 (IL-22) production by innate lymphoid cells 3 (ILC3), an important cytokine for inflammation regulation [48]. To further understand the role of the bacterial metabolite, mice were treated with a diet lacking Trp. The results were a changed gut microbiome community and impaired intestinal immunity, suggesting that the Trp metabolism is an important regulatory element [48]. However, the source and nature of the Ahr ligands remain unclear, and more research is required.

Insulin-like growth factor 1 (IGF-1) was the first hormone linked to the gut-bone axis. When colonizing germ-free (GF) mice, which lack a gut microbiome, with specific pathogen-free mice (SPF), an analysis of bone formation and resorption revealed an increase [49]. Furthermore, serum levels of IGF-1 substantially increased in colonized mice, with production reported in the liver and adipose tissue. In contrast, antibiotic treatment reducing the microbiome of conventional mice reported decreased IGF-1 production and less bone formation [49].

Hydrogen sulfide (H_2_S) has been suggested as another bone-regulating molecule generated by gastrointestinal cells and by bacteria within the gut [47]. Germ-free mice were reported to have low serum and gastrointestinal levels of H_2_S, implying that the microbiota accounts for a considerable amount of H_2_S blood levels in the animal model [47]. Abnormal levels of hydrogen sulfide have been associated with deficiencies in bone homeostasis by causing an influx of calcium into cells due to a reduction of sulfhydration of cysteine residues in calcium TRP ion channels [50]. The decrease in calcium flux downregulates protein-kinase-mediated pathways that control the differentiation of bone marrow mesenchymal stem cells, which can differentiate into osteoclasts and osteoblasts [51]. Various current genome-wide association studies based on Mendelian randomized approaches have identified the taxa *Clostridiales* and *Lachnospiraceae* associated with bone mass alteration [29]. In a series of clinical trials involving181 participants, abundant levels of *Clostridium Cluster XIVa* (a genus of the *Clostridiales* class belonging to the *Firmicutes* phylum) were reported in two-thirds of the subjects that had skeletal conditions of osteopenia or osteoporosis versus subjects with normal bone mass density [29]. Similarly, among 161 postmenopausal women, those with postmenopausal osteoporosis and/or osteopenia presented lower richness and diversity of the gut bacterial community, as well as higher relative abundances of *Lachnospira pectinoschiza*, a species belonging to the *Lachnospiraceae* family within the *Firmicutes* phylum [29].

## Mechanisms of gut-microbiota-bone interaction

### Inflammation

The more recent understanding of the bone remodeling process has established a correlation between factors involved in inflammation and bone physiology. It is well documented that the increased production of pro-inflammatory cytokines while growing in age is related to the escalation in osteoporosis cases in the elderly [6]. In fact, recent data suggest that bone erosion seen in conditions such as osteoporosis and relevant inflammation are enhanced by the same immune component [5]. Cytokines released by immune cells that are pro-osteoclastic stimulate osteoclast differentiation and/or activity. Moreover, immune cells and bone cells interact in diverse ways within the bone microenvironment. Bone and T-cells share the same progenitors residing in the bone marrow, and they are influenced by several of the same cytokines [52]. Other immune cells from the humoral response, such as B-cells, and the innate response, such as macrophages and natural killer cells, have been shown to influence osteocytes. Thus, T-cells produce and respond to inflammatory cytokines, including interleukins (IL) -2, -4, -6, -17, tumor necrosis factor alpha (TNF-α), transforming growth factor beta (TGF-β), and interferon gamma (IFN-γ). Interestingly, osteoclasts can respond to pro-inflammatory IL-17 and TNF-α produced by T-cells, increasing their activation and survival [53, 54]. Given the connection between the gut microbiome and immune system, we suggest that dysbiosis in the gut may influence the differentiation of T-cells, leading to a pro-inflammatory response. This, in turn, activates osteoclasts, disrupts bone remodeling, and ultimately results in bone loss. In sterile mice, monocyte and osteoclast presence diminishes in the bone marrow, whereas their levels are restored upon the reintroduction of gut microbiota [5]. The interplay between Th-17 cells and T-reg cells is crucial for the regulation of inflammation, and the gut microbiome may exert a direct effect on the presence of immune cells. It is suggested that the gut microbes contribute to the cell signaling, however, whether or which certain bacterial species directly impact immune cell signaling and immune cell differentiation requires further investigation. In a study by Donkor et al. [55], macrophages and mononucleated cells from the umbilical cord and spleen were stimulated with probiotics *Bifidobacteria* and *Lactobacillus reuteri* among others. An increase in pro-inflammatory and anti-inflammatory cytokines was reported; noticeably large amounts TGF-β inducing the differentiation of T-reg cells, demonstrating that gut bacteria can alter the inflammatory response of certain immune cells. Immune response in the intestine is mainly mediated by peripheral blood T-reg cells (pT-reg), and its main function is to control pro-inflammatory response of food factors, as well as maintaining a rich microbial community for digestion. As there are more bacteria in the colon, there is also a higher concentration of pT-reg cells there. In the colon, studies have shown that *Clostridium* [56], *Bacteroides, Bifidobacterium, Lactobacillus, *and *Helicobacter* [57] use bacterial components to promote the production of pT-reg cells by activating the innate immune receptor TLR2 [5]. Other bacteria like *H. hepaticus* also signal through TLR2 mentioned above and induce an anti-inflammatory cascade signal that makes macrophages release IL-10. Actually, IL-10 production by T-reg cells seems to be very dependent on the gut microbiota since sterile mice had a low concentration of IL-10 producing T-regs, and colonization of those sterile mice with *Clostridium* or *Bacteroides fragilis* significantly increased the abundance of IL-10 producing T-regs [5]. Additionally, colonization enhanced the expression of the CTLA-4 receptor in T-regs that downregulates the inflammatory response [56].

We have synthesized the evidence that gut bacteria regulate immune cells and inflammation responses by influencing cell signaling proteins and immune cell activity with bacterial metabolites. We have also demonstrated that these mechanisms are related to bone physiology and remodeling. Therefore, since our gut bacteria maintain anti-inflammatory processes, dysbiosis and loss of diversity could remove these anti-inflammatory processes and induce bone loss, perhaps promoting osteoporosis.

### Short-Chain Fatty Acids

The intestinal microbiota can regulate various host functions, including metabolism [58, 59], immunity [60], brain function [61], and bone metabolism [62]. Microbial metabolites such as Short-Chain Fatty Acids (SCFAs) are important mediators of these interactions. The primary short-chain fatty acids in the intestine are butyrate, propionate, and acetate, occurring in a ratio of 60:20:20 by mole, respectively [63]. Another way that the gut microbiome may regulate the inflammatory response through T-regs is with SCFA metabolites. SCFAs are produced through the fermentation of resistant dietary carbohydrates, such as dietary fiber, by the gut bacteria [64]. On the topic of diet, fiber, and omega-3-rich diets increase the total production of SCFA, and these fatty acids have directly been linked to benefit cardio-metabolic health as well as improving the gut barrier integrity, regulating the immune system, and reducing inflammation [64]. There is ample research demonstrating that SCFAs promote pT-reg cell responses in the gut and colon [65]. More specifically, SCFA metabolites like butyrate and niacin are thought to regulate T-regs by being recognized by G-protein receptors GPR43 and GPR109A on the cell surface, triggering a signal cascade through secondary messenger proteins, activating the cell, and suppressing colonic inflammation [66]. SCAFs can cross the intestinal barrier by free diffusion, carrier-mediated transporters, or binding to G protein-coupled receptors GPR41 and GPR43 [67]. Once in the bloodstream, they can reach different organs, including bone, where they can directly affect cells involved in the fracture healing process [67]. Using Germ-Free mice, Lucas et al. [68], in a series of experiments with direct SCFA supplementation, high-fiber diet, and bacterial transfer, found consistent beneficial effects of SCFA levels on bone homeostasis. The authors attribute the protective roles of SCFAs on bone loss mainly to the suppression of osteoclast differentiation and function. The proposed mechanism of suppressing osteoclast differentiation mediated by butyrate involves the expansion of regulatory T (Treg) cells, triggering the release of Wnt10b by CD8+ T cells and consequent activation of Wnt signaling in osteoblasts [69]. The conversion of soluble fiber into short-chain fatty acids (SCFAs) also lowers the gut’s pH, contributing to calcium uptake and other minerals important for healthy bone, such as potassium, and inhibiting the formation of osteoclasts [70].

### Gut barrier integrity

In a healthy individual, the intestinal epithelial barrier is highly selective to the luminal contents and does not permit the passage of microbial organisms or antigens. This physical barrier is enforced by a thick mucus layer lining the colonic epithelium and creating separation between microbes and enterocytes as well as paracellular tight junctions (TJ). This viscoelastic substance is structurally supported by heavily O-glycosylated mucin proteins, which act as a biological sieve and constitute only 1-5% of the aqueous gel [71, 72]. In mouse models, a healthy inner mucus layer has pore sizes down to 0.5 μm, which is not penetrable to bacteria [73]. While the mucosal layer prevents microbial invasion, it forms a diffusion barrier in which small molecules, such as ions, water, nutrients, and gases, can readily diffuse through it and reach the epithelium [74].

There are currently 21 MUC genes identified in humans, with 15 expressed and secreted in various regions of the GI tract by goblet and paneth cells [75-77]. Their unique glycosylation profiles allow for selection of particular commensal bacteria which reside in or degrade mucus such as *Akkermansia muciniphila* [78]. Mucins also constrain the immunogenicity of bacterial antigens and prevent inflammation by inducing dendritic cell-conditioning signals in the intestinal epithelium [79]. Muc2 glycans reduces expression of pro-inflammatory cytokines IL-6, IL-8, IL-12, and TNF-α in response to toll like receptor ligands such as LPS, flagellin, or TNF while enhancing expression of the anti-inflammatory cytokine IL-10 [79]. As such, Muc2 mediates gut homeostasis of resident microbiota by delivering tolerogenic signals to dendritic cells.

However, a compromised mucus layer leaves mucosal membranes, especially the gut, at risk of a weakened defense against infections, increased inflammation, and damaged epithelial tissue. An increase in intestinal permeability might result in the translocation of bacteria or its microbial products (e.g., lipopoly-saccharides) to the bloodstream, activating and prolonging osteoclastogenesis through the release of pro-inflammatory cytokines [53, 54, 80]. The integrity of the mucosal structure is influenced by various factors such as microbiome, diet, circadian rhythm, stress, pH, CaCl_2 _balance, and most noteworthy, age [26, 36, 81-84]. As mice age into an elderly stage, low-grade inflammation is common and often accompanied with a decline in the mucus barrier as well as commensal *A. muciniphila* [82-84]. There is much to uncover about the link between the intestinal barrier and osteoporosis, however, exploring mucin as a potential therapeutic target may aid immune tolerance, reduce bacteria translocation from the lumen into the intestinal epithelium and consequently, ameliorate bone loss.

## Current studies on the association between gut dysbiosis and osteoporosis

First, it is important to emphasize that to claim an association between gut microbiome and osteoporosis, additional research with larger sample sizes, better controls, and concise results is required. The aim of this paper is to develop an interest in allocating resources to pursue knowledge of the gut-bone axis mechanisms since there are not enough studies to draw conclusions. Second, we can only suggest an association based on our understanding of the interactions between the bacteria and our immune system. Bone mass density (BMD) (usually of the spine) is the basis for osteoporosis diagnosis, from which the severity of the disease can be determined. In a first study by Xu et al. [8], relationships between bone mass density and microbiota in osteoporosis patients were observed using weighted Unifrac, relative abundance, and PCA plots. At the phylum level, *Euryarchaeota* and *Actinobacteria* were negatively correlated with BMD in the hips. At the genus level, *Haemophilus* and *Bifidobacteria* were negatively correlated with BMD in the spine and hips. Unfortunately, there was no significant difference in the microbes at each taxonomic level between osteoporosis patients (OP) and healthy controls (HC), and no correlation was found between the Alpha diversity of the microbiome and BMD [8]. However, in a following study by the same researchers, it was found that the abundance of microbes in OP was quite higher than in HC, suggesting that an excessive increase in gut bacteria leads to bone loss. The question arises as to how this is possible if we previously determined that good gut bacteria have anti-inflammation properties. Would increasing the number of bacteria not be strictly beneficial? Our research across all these studies has demonstrated that healthy gut diversity is delicate and must be precisely balanced. When the ratios of gut bacteria fluctuate substantially, dysbiosis occurs. *Faecalibacteria* and *Dialister* genera were reported in higher abundance in OP. However, *Faecalibacteria* produces butyric acid SCFA and promotes bone formation, so their excessive abundance does not cause bone loss. Still, it could be a sign of feedback regulation of the system due to the decrease in bone mineral density of the host [85]. Additionally, *Dialister* abundance may be related to decreased alveolar bone mass [86]. Thus, microbe density increases in OP, but functional modules such as membrane transport and carbohydrate metabolism were significantly reduced. Disorders in membrane transport affect solute exchange between internal and external regions, affecting the metabolism of calcium salts [8].

In a study by Ling et al. [87], no significant differences in alpha diversity were reported between HC and OP, indicating an even distribution of microbiota in the samples, similar to the result obtained in the study by Xu et al. [8]. However, the change in beta diversity, or the degree of community differentiation, showed that the gut communities changed between OP and HC. At the lumbar spine, enrichment of *Streptophyta* and *Phascolarcto-bacterium* was associated with the presence of osteoporosis. At the femoral neck, enrichment of *Actinobacillus*, *Blautia, Oscillospira, Eggerthella, Rikenellaceae, Phascolarctobacterium*, and *Bacteroides* was associated with osteoporosis. Lower enrichment of *Ruminococcaceae, Collinsella, *and *Veillonellaceae* was associated with osteoporosis in the femoral neck [87]. Unfortunately, in most cases, partial 16S rRNA gene sequencing cannot distinguish microbes at the species level, which results in findings that lack specificity. Although the results obtained require more attention to detail to establish an accurate relationship between gut microbiota, cytokines, and bone cells, it has been demonstrated through sequencing that bacterial communities do differ greatly between OP and HC. Once we acquire the technology to differentiate the bacteria at a species level, we hypothesize that the results will be even more relevant and tell which mechanisms are employed.

In a study performed by Wang et al. [88], in Xi’an, China, 12 osteoporotic subjects showed higher proportions of *Blautia* and *Parabacteroides* but lower proportions of *Ruminococcaceae* compared to 6 healthy controls. In a study done by Das et al. [89], in Ireland, *Actinomycetes, Eggerthella, Clostridium X1Va*, and *Lactobacilli* were more abundant in 61 osteoporotic patients compared to 60 healthy controls. These studies have demonstrated that bone mineral density is dependent on pathways in the gut, such as lipopolysaccharides biosynthesis and membrane transport, which aligns with work done by Xu Z, et. al [8]. Moreover, the analysis of fecal metabolites such as N-acetylmannosamine, deoxyadenosine, and adenosine may provide insights into low bone density within the body [87]. Despite these discoveries, there is substantial room for improvement and potential for criticism. Given the significant impact of environmental factors on an individual’s gut microbiota, it is difficult to directly compare the aforementioned studies. Dietary habits in Ireland and China differ considerably, and the comparison of microbiome diversity among different populations poses a challenge. Available medicine and antibiotics that impact the bacteria can also differ from one continent to the next. Additionally, the studies were executed with a very low number of subjects which indicates that the results obtained may not accurately represent a wider population. Identifying biomarkers related to osteoporosis in the gut is difficult due to the complexity of the gut microbiome, the current limitations in resolving specific species, and the limited understanding of bone-health related biomarkers. While these studies contribute to solidifying the association between the gut microbiome and osteoporosis, the causal nature remains unclear, largely due to the knowledge gaps and limited resources in this field.

## Bone loss in space travelers

Space travelers experience bone loss at a rate of 1-1.5% of total bone density per month [90]. Anti-resorptive bisphosphonates are currently being employed to reduce bone loss in-flight, but they can interfere heavily with post-flight recovery, sometimes rendering it incomplete [91]. Therefore, microgravity induced bone-loss is an unresolved problem for astronauts, and the contribution of different processes to bone loss remains unclear. What has been demonstrated is that the environment is a significant factor for changes in the gut microbiome, and it is suggested that the micro-gravity environment in space could lead to dysbiosis of those bacteria. Thus, finding a way to maintain a healthy gut microbiome could reduce the amount of bone loss in space flight.

Recorded in-flight changes were reported to be a decrease in calcium homeostasis stress regulators. For example, the parathyroid hormone (PTH), 1,25- dihydroxyvitamin D, and calcitonin decreased by 11-23% during early spaceflight [91]. Additionally, bone formation markers decreased at an exponential rate during flight. On the other hand, bone resorption markers increased by 113% within 11 days of flight. Upon return to Earth, the calcium regulators increased linearly, and bone formation markers increased at a linear rate of 84% per month [91]. Interestingly, although bone loss in limbs was progressive throughout space travel, long-duration missions reported less bone loss than shorter-duration missions, suggesting that microgravity-induced bone loss may diminish over time. The crews participating in longer space voyages also had access to advanced nutrition and exercise. Nonetheless, these methods do not completely negate bone loss in space, and bone density increase was observed in the skull, which is mechanically neutral, suggesting that nutrition may play a large role in maintaining a healthy bone physiology [91].

We believe that there is a possibility for a lead on whether the gut microbiome may have an impact on bone loss in space travel. In a study by Voorhies et al. [92], researchers investigated the impact of lengthy space travel durations on the gut microbiome of 9 astronauts over 6 to 12 months aboard the International Space Station. Like the microbial changes between osteoporosis patients and healthy controls, microbial communities in the gut of these astronauts changed while in flight, supposedly due to the change in environment. It was found that alpha diversity, or the diversity of species at a local scale, significantly increased while in space and returned to normal levels once back on Earth [92]. There was no significant change in beta diversity. In space, 17 genera of bacteria inside the GI tract reported a change in abundance in collected samples. Out of these 17 genera, 13 belonged to the *Firmicutes* phylum and displayed an increase in abundance, mainly in bacteria of the order *Clostridiales*. Additionally, while in space, there was a reduction in the relative abundance of three bacterial genera known for anti-inflammatory properties: *Akkermansia*, *Fusicatenibacter, and Pseudobutyrivibrio* [92]. These compositional changes generally reverted to normal once back on Earth.

Cytokine abundance was also subject to change during space travel. Changes in cytokine abundance in the plasma compared to changes in the bacterial composition of the gut were observed in the Voorhies et al. [92] study. Ten significant changes to cytokine composition were reported compared to pre-flight values. After 180 days of flight, an increase in pro-inflammatory cytokines MCP-1, IL-8, IL-1b and MIP-1β, and a rise in TNF-α was reported. Elevated infight levels of cytokines IL-2 and IL-1ra were also detected [92]. All these values returned to pre-flight levels once back on Earth. The most notable correlation between the increase in pro-inflammatory cytokines and the involvement of the gut microbiome was a report that the abundance of 16S rRNA sequences containing a certain percentage of divergence (OTU000010) of the genus *Fusicatenibacter* was negatively correlated with the concentration of pro-inflammatory cytokines IL-8, IL-1b, IL4, and TNF-α. Changes in OTU000011 of the genus *Dorea* were also negatively correlated with changes in the level of IL-1b, IL-1ra VEGF, and MIP-1b that showed an increase in space [92]. Comparable to the challenges discussed when studying the gut microbiome on Earth, a larger sample of astronauts would be required to obtain more concise results. More opportunities for rigorous and long-duration research with a higher frequency are also required to obtain more compelling data.

## Final remarks

Three gut-microbiome-changing factors were identified at the start of this study: age, diet, and environment. Changes in the diversity and richness of the bacteria in the human gut were observed when these factors fluctuated, however, results were inconsistent across studies. In a clinical setting, it was demonstrated throughout this paper that the bacteria in our gut change when comparing healthy controls and those experiencing bone loss. Additionally, the release of anti-inflammatory cytokines is thought to be associated with certain bacteria, and metabolites from gut bacteria are thought to aid the immune system and regulate bone density. Nonetheless, identifying specific species and the mechanism of their impact on bone density and inflammation remains greatly unknown.

**Figure 1.**
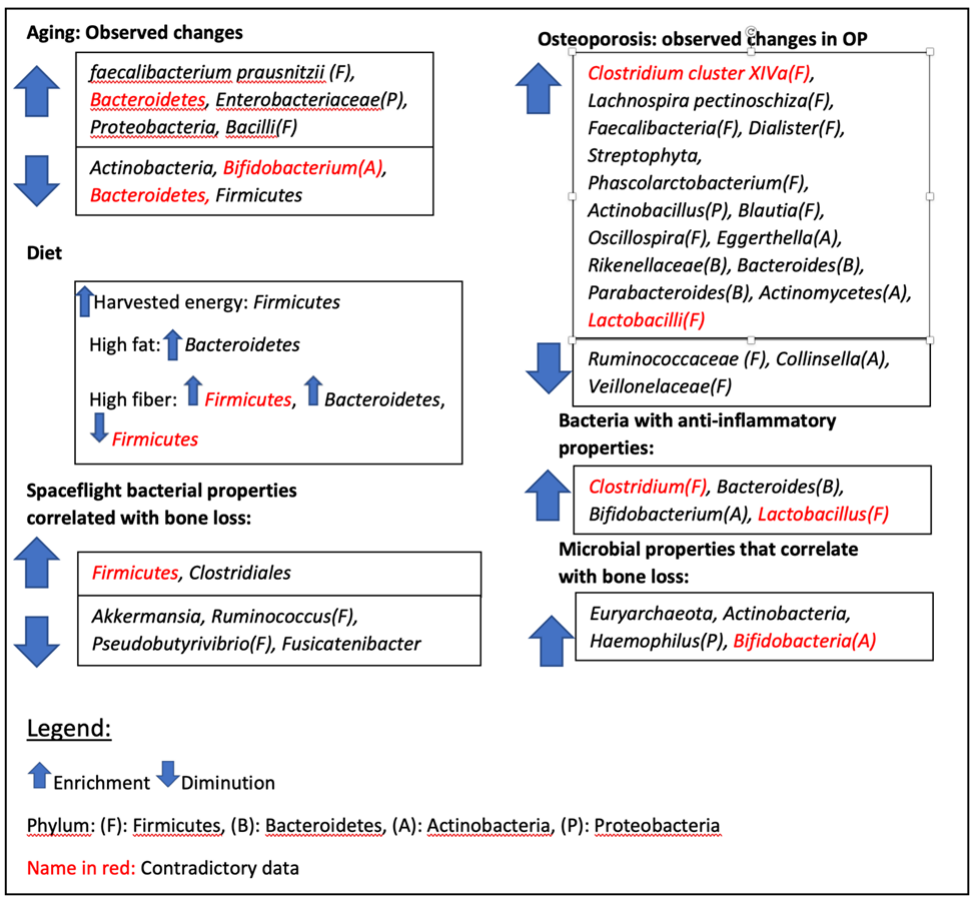
Summary of our findings.

Research on the gut microbiome is currently challenging. Studies on humans were effective in demonstrating that certain bacteria with specific genes for nutrient breakdown are present only in certain populations based on their diet or environment. It is hard to establish a baseline for a healthy gut since different cultures around the world have different healthy gut microbiota. It is also hard to understand how bacteria interact with the host in their natural environment. Most of the data is collected on fecal samples containing bacteria that have lost all the information about their niche environment in the intestine. Thus, the interdependence between bacteria sharing the same niche in the intestine is lost. It is currently challenging to pinpoint in detail which species of bacteria is responsible for which pathway, so results tend to be vague. Finally, many studies give contradicting results, have conflicting controls, omit factors that would influence their data, and remain vague with the specificity of the taxa. Hence, many of the studies analyzed for this paper contained comparisons of the gut microbiome at the Phylum level. These studies did not answer which bacteria affect bone physiology in the host, and the data on bacterial ratios is apprehensive but not specific. Studies on animal models were effective in demonstrating that providing a gut microbiome to a sterile mouse changes bone regulation, cytokine release, inflammation regulation, and the host immune system response. Hence, we know that the gut microbiome changes between healthy controls and subjects experiencing bone loss, whether it is due to their increasing age, altering diet, or changes in the environment, like during spaceflight. At present, it cannot be determined which increase in species may be responsible for beneficial changes in host metabolisms. A summary of our findings can be found in Figure 1. As a matter of fact, it has often been suggested throughout the studies that an increase or decrease of a gut bacterium may result in dysbiosis, even if that bacterium is beneficial to the host. The concentration of gut bacteria for a healthy microbiome tends to be precisely measured, and any substantial alterations seem detrimental.

## Implications for clinical practice and future research

There have been insufficient studies, especially in the Western region, addressing treating inflammation and bone loss. Exploring gut health intervention without severe side effects associated with current pharmaceutical approaches may offer a natural and effective alternative to confront bone loss and inflammation, especially when considering the substantial annual cost for osteoporosis medication in the US. Investigating the correlation between gut microbiota dysbiosis and primary osteoporosis could provide insights for preventive and nutritional prebiotic or probiotic treatment. Such interventions could also improve astronauts’ health, enhance their quality of life, and reduce disability in the general population.
